# Evaluation of Therapeutic Benefits of a Novel Herbal Ingredient-Based Hyaluronic Acid Gel as Monotherapy for Inflammatory Enlargement of a Patient with Diabetes Mellitus

**DOI:** 10.1155/2022/4872959

**Published:** 2022-02-04

**Authors:** Jaahnavi Lanka, Santhosh Kumar

**Affiliations:** Department of Periodontology, Manipal College of Dental Sciences, Manipal, Manipal Academy of Higher Education, Manipal, 576104, Udupi, Karnataka, India

## Abstract

The reason for the destruction of the attachment apparatus in patients with periodontal disease is the supra- and the subgingival microflora. Hence, the treatment of this inflammatory gingival disease is primarily focused on eliminating the cause. The present case report assessed the therapeutic benefit of adjunctive use of a commercially available topical agent containing 1% hyaluronic acid gel combined with nonsurgical periodontal therapy. A patient aged 64 years with type 2 diabetes mellitus reported to the Department of Periodontology. He complained of gum enlargement and frequent bleeding during brushing. The patient was not under any medication for his diabetes control. On examination, there was a localized papillary gingival enlargement. Therefore, Klirich gel containing hyaluronic acid was applied on the surface of the gingiva during the first visit of the patient. During the follow-up visit, he continued with this adjunctive use of the gel along with the nonsurgical periodontal therapy. In addition, he controlled his blood sugar level with diet and exercise. During the follow-up visit after one year, there was a noticeable clinical change in the overall health of his gingiva.

## 1. Introduction

Gingival enlargements occur due to excessive proliferation of gingival soft tissues [1]. The abnormal increase in the size of the gingiva can cause functional and aesthetic concerns to the patient. The etiology can be local factors like plaque and calculus or systemic diseases [2]. Malignant etiologies like hematological and squamous cell carcinoma should be ruled out. Other etiologies include periodontal disease, genetic diseases, conditioned enlargements like puberty, pregnancy, and systemic diseases, and drug-induced and iatrogenic causes [3]. A few drugs that are studied to cause gingival enlargement are cyclosporin, phenytoin, and calcium channel blockers [4]. Bondon-Guitton and colleagues [5] in 2012 studied that a few other drugs like immunosuppressants, anticonvulsants, antibiotics, and contraceptive pills also caused gingival enlargement.

In cases of drug-induced gingival enlargement, replacing the causative drug resolves the enlargement [6] in most cases. In other cases presenting with unknown etiology, thorough scaling and root planing will be needed to manage the local factors. If the enlargement does not subside after nonsurgical therapy, surgical procedures like gingivoplasty and gingivectomy can eliminate pockets and improve oral hygiene [7, 8].

Evidence from human studies suggests that gingival enlargement is mainly influenced by the microbial aspect [9, 10], which forms the basis for the use of antimicrobial therapy as an adjunct in the management of gingival enlargement. The outcomes of periodontal debridement have been enhanced by the adjunctive use of systemic antibiotics, topical antibiotics, and topical antiseptics [10]. However, adverse reactions, hypersensitivity, superinfection, and antibiotic resistance have been widely reported with antibiotic use. Considering the various shortcomings of antimicrobial therapy, the need for the development of therapeutic agents which are safe and biocompatible is undoubtedly high. Hence, research on plant-based herbal agents came into existence. Natural therapeutic agents such as antioxidants, micronutrients, and herbal medications have been proposed and tested [11, 12]. Herbal agents are very effective and comparable to antimicrobial therapy in managing various periodontal conditions.

In the present case report, the therapeutic benefit of Klirich® (Itena Clinical products, France) containing 1% high molecular weight hyaluronic acid gel in combination with herbal ingredients like alchemilla leaf extract, calendula flower extracts, avocado oil, clove essential oil, and grapefruit seed extract given as monotherapy is briefly illustrated and examined in a subject with gingival inflammation and diffuse enlargement secondary to uncontrolled diabetes mellitus.

## 2. Case Report

**Figure 1:**
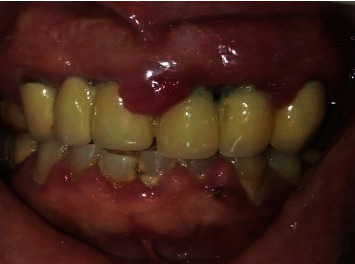
Presentation of gingival enlargement at baseline.

**Figure 2:**
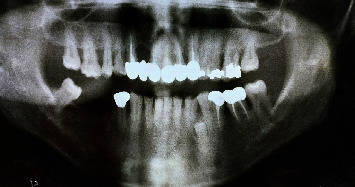
Preoperative radiograph.

In 2019, a male patient aged 65 years reported to the Department of Periodontology, Dental Outpatient ward, with a chief complaint of swelling around the gums and bleeding during brushing. Upon clinical examination, gingival enlargement was observed in the upper and lower anterior tooth regions (Figure 1), and there was a generalized, moderate (up to the coronal third of the root) to severe (up to the middle third of the root) bone loss in the panoramic radiograph (Planmeca Promax, Finland, Romexis software) (Figure 2). The patient gave a history of bleeding gums while brushing his teeth and discomfort due to swollen gums. His systemic health history was recorded, and the patient was a known case of diabetes mellitus which was not under medication at the initial visit. All the possible etiologies were ruled out by a thorough case history, blood investigations, and biopsy. The fasting blood glucose level was 145 mg/dl at the initial visit. The patient's past dental history was uneventful, where he underwent procedures ranging from oral prophylaxis and periodontal flap surgery four years from the presenting complaint. The patient presented with a prosthesis in the upper anterior region from 13 to 23. The enlargement could be easily distinguished from the surrounding gingiva. The patient had difficulty in oral hygiene maintenance due to the enlargement. The proper placement of restorative margins and cleanability were evaluated to rule out any possible irritation from the restorative materials from the fixed prosthesis [13]. Periodontal pocket was recorded using the Hufriedy (Chicago, United States) University of North Carolina-15 (UNC-15) probe. The pocket charting showed persistent pocket depths ranging from 5 mm to 6 mm, in the upper anterior and the posterior region suggesting moderate attachment loss. Poor oral hygiene maintenance by the patient and the overhang surrounding the fixed prosthesis could have resulted in the accumulation of the deposits. The case was diagnosed as generalized stage II grade B periodontitis, currently unstable with a risk factor of diabetes mellitus. The gingival inflammation and the enlargement were secondary to uncontrolled diabetes mellitus, plaque, and calculus deposits.

### 2.1. Treatment Planning

**Figure 3:**
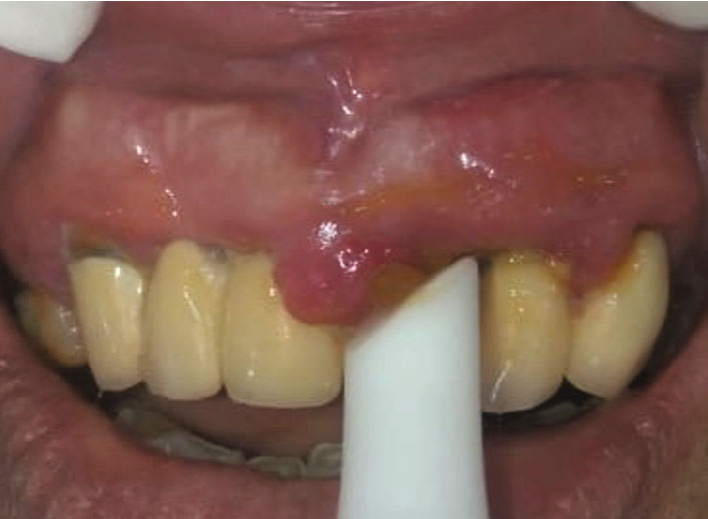
Application of Klirich gel on the inflamed areas as monotherapy.

The treatment plan was a comprehensive periodontal therapy comprising a nonsurgical phase, maintenance phase, and surgical therapy. However, in the first visit, scaling was postponed due to increased blood sugar levels (fasting blood sugar:145 mg/dl), and the application of Klirich gel, a patented formula of *Itena Clinical products*, *France* (*EP2954902 A1*), was performed as monotherapy, as seen in Figure 3. The application of the novel gel as monotherapy was made considering the available evidence from studies reporting management of oral lesions in uncontrolled diabetic patients by using plant-based gels like aloe vera as monotherapy, which showed beneficial results [14]. The instructions of use are illustrated in Table 1. The patient was referred to a physician for a review of diabetes mellitus and suggested to come back after the blood sugar levels returned to normal. In addition, the patient was educated about the importance of oral hygiene, and brushing technique was demonstrated. A review after two weeks was scheduled. At this review, fasting blood sugar levels were found to be 128 mg/dl.

### 2.2. Nonsurgical Therapy

**Figure 4:**
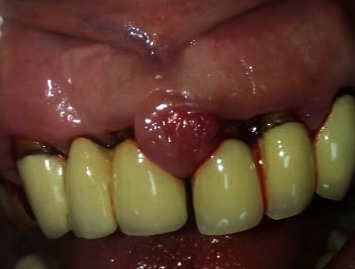
Review at 2 weeks: subsided inflammation, except for the interdental area.

After two weeks of monotherapy, with Klirich gel (no scaling has been performed during the first two weeks), grade II (Bökenkamp and Bohnhorst index 1994 [15]) overgrowth resolved, as seen in Figure 4. However, the enlargement in the interdental area between the two upper central was persistent, and when palpated, it was a fibrous nodular overgrowth, which was later diagnosed as pyogenic granuloma after a biopsy.

At the second visit, supragingival, subgingival scaling, root planing, and curettage were performed. Klirich gel was given for topical application again as described previously, and the patient was recalled for review after two weeks.

### 2.3. Surgical Therapy

**Figure 5:**
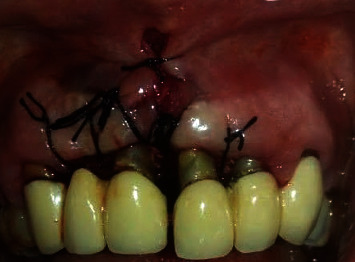
Surgical phase: 4 weeks: gingivectomy and open flap debridement.

At the four-week review, as the upper anterior region showed persistent probing pocket depths, it required surgical intervention. For the surgical excision of the gingival enlargement, an internal bevel gingivectomy of the fibrotic overgrowth and open flap debridement were planned. Initial probing and the marking of the periodontal pocket depth were done using the pocket marker (Crane Kaplan, Hufriedy). Then, an internal bevel gingivectomy was performed using the Kirkland knife and Orban's interdental knives. Residual tissue tags were cleaned, and the margins were smoothened using the no. 15 B. P. blade and handle (Figure 5). The 4-0 silk sutures were used for approximation of the flaps. There was a high frenal attachment and needed management. Hence, conventional frenotomy was performed to remove the aberrant frenum. Analgesic ibuprofen 400 mg (Brufen®) was prescribed and instructed to be taken thrice daily for two days till the pain subsided, and postoperative instructions were given.

**Figure 6:**
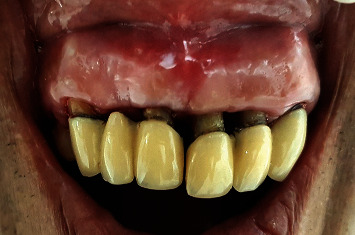
Postoperative clinical presentation after complete healing; resolved gingival enlargement and reduced pocket depths at 1-month postoperative review.

**Figure 7:**
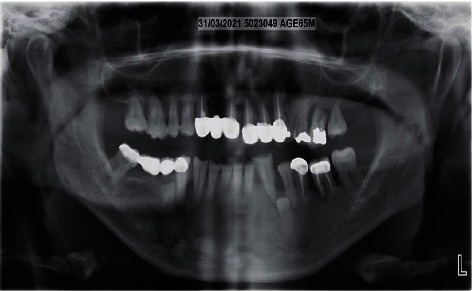
Postoperative radiograph after one year.

During the postoperative visit, the healing was uneventful, the enlargement was eliminated, and pocket-depth reduction was noted when evaluated at a one-month review (Figure 6). The postoperative radiograph after one year is shown in Figure 7.

## 3. Discussion

Periodontal disease management has always been challenging in diabetic patients. Research in these aspects indicated that periodontal disease and diabetes have a synergistic relationship [16–19]. The risk of periodontal disease aggravated systemic disorders like diabetes mellitus [19]. Difficulties posed by wound healing patterns in diabetic patients also restricted periodontal management to nonsurgical therapy [16–19]. However, the fact that deeper bone defects or residual pockets cannot be eliminated by nonsurgical therapy alone makes surgical intervention necessary in these areas. Hence, adjuvants to surgical therapies that enhance wound healing are invaluable for better prognosis and long-term success in periodontal management. Zhang et al. [20] observed that hyaluronic acid-based hydrogel shows anti-inflammatory effects and promotes angiogenesis in chronic diabetic wounds. The use of hyaluronic acid in combination with nanofibers for enhanced wound healing was studied by El-Aassar and his colleagues in January 2021 [21].

The bacteriostatic property of hyaluronic acid is also well established by a study conducted by Zamboni et al. [22]. In a recent animal study by Shirakata et al. [23], periodontal management of infrabony defects using hyaluronic acid has shown beneficial results. Mamajiwala and coworkers [24] have seen improved clinical and radiographic outcomes using 0.8% hyaluronic acid as an adjunct in treating periodontal infrabony defects by open flap debridement.

Besides the excellent wound-healing abilities of hyaluronic acid, its bacteriostatic activity, biocompatibility, nonimmunogenic property, and safety were confirmed by Becker et al. [25] and various other studies [20–26], suggesting that hyaluronic acid is an ideal adjuvant in periodontal surgical and nonsurgical therapy.

The FDA approved it in 2005, which also published a summary of the safety and effectiveness data in the same year [27]. Hence, in the present case report, the use of herbal ingredients in combination with hyaluronic acid has been evaluated. The combination of all the herbal agents with hyaluronic acid in the present case showed a remarkable reduction in inflammation and swelling. However, biochemical analysis of inflammatory biomarkers would have added more scientific evidence to the case report.

A larger group randomized trial could provide evidence of the effectiveness of this gel. Further comparison with new alternative treatment options, like probiotics [28], antioxidants [29], and plant-based therapy [30], could be assessed in patients with periodontal disease. Apart from these alternatives, using lasers to manage gingival inflammation and enlargement is currently being studied as an effective treatment option [31].

## 4. Conclusion

The use of Klirich ® in the present case has shown a therapeutic benefit as an adjunct to scaling and root planing. It is clinically safe and effectively manages inflammatory gingival enlargement due to periodontal disease in diabetes. Furthermore, the present study's findings should be confirmed in future randomized controlled clinical trials, including microbial and biochemical analysis.

## Figures and Tables

**Table 1:** Ingredients of Klirich gel.

Nonanimal-based herbal ingredients	Main ingredient
Grapefruit seed extract Alchemilla leaf extract Clove essential oil Avocado oil Calendula flower extract	1% high molecular weight hyaluronic acid
Instructions for usage (i) Massage once daily after brushing (ii) Apply a pea-sized amount of gel on the inflamed areas and massage the gel with a clean finger for 15 seconds until it gets absorbed (iii) Let the gel stay in the mouth for another 15 seconds without massaging (iv) Recommended period of use can range from 1 to 2 weeks and may vary depending upon the severity of inflammation	

## Data Availability

All the data is included in the main text.

## References

[1] Korczeniewska O. A., Hart T. C., Diehl S. R. (2021). The role of genetics in oral medicine. *Burket's Oral Medicine.*.

[2] Punj A., Chaturvedi M. (2020). Diagnosis and treatment plan for gingival diseases and conditions. *Oral Diseases*.

[3] Agrawal A. A. (2015). Gingival enlargements: differential diagnosis and review of literature. *World Journal of Clinical Cases*.

[4] Hatahira H., Abe J., Hane Y. (2017). Drug-induced gingival hyperplasia: a retrospective study using spontaneous reporting system databases. *Journal of Pharmaceutical Health Care and Sciences*.

[5] Bondon-Guitton E., Bagheri H., Montastruc J. L. (2012). Drug-induced gingival overgrowth: a study in the French Pharmacovigilance Database. *Journal of Clinical Periodontology*.

[6] Bharti V., Bansal C. (2013). Drug-induced gingival overgrowth: the nemesis of gingiva unravelled. *Journal of Indian Society of Periodontology*.

[7] Feijó Miguelis T. M., von Ahn Pinto K., Rilling Nova Cruz L. E., Aitken Saavedra J. P., Martos J. (2019). Systemic and clinical treatment of gingival hyperplasia associated with use of anticonvulsant. *Revista Clínica de Periodoncia, Implantología y Rehabilitación Oral*.

[8] Khera P., Zirwas M. J., English J. C. (2005). Diffuse gingival enlargement. *Journal of the American Academy of Dermatology*.

[9] Bontemps L., Gaultier F., Anagnostou F., Ejeil A. L., Dridi S. M. (2021). Drug-induced gingival overgrowth. *Drug-Induced Oral Complications*.

[10] Mawardi H., Alsubhi A., Salem N. (2021). Management of medication-induced gingival hyperplasia: a systematic review. *Oral Surgery, Oral Medicine, Oral Pathology, Oral Radiology*.

[11] Muralidharan S., Giri M., Patankar S., Kamble A. P., Kamble D. A., Ismail N. (2021). The effect of triphala and chlorhexidine mouthwash on dental plaque and gingival inflammation: an open labelled clinical study. *Natural Volatiles and Essential Oils*.

[12] Mazur M., Ndokaj A., Jedlinski M., Ardan R., Bietolini S., Ottolenghi L. (2021). Impact of green tea (*Camellia sinensis*) on periodontitis and caries. Systematic review and meta-analysis. *Japanese Dental Science Review*.

[13] Avetisyan A., Markaryan M., Rokaya D. (2021). Characteristics of periodontal tissues in prosthetic treatment with fixed dental prostheses. *Molecules*.

[14] Ghasemi N., Behnezhad M., Asgharzadeh M., Zeinalzadeh E., Kafil H. S. (2020). Antibacterial properties of Aloe vera on intracanal medicaments against *Enterococcus faecalis* biofilm at different stages of development. *International Journal of Dentistry*.

[15] Bökenkamp A., Bohnhorst B., Beier C., Albers N., Offner G., Brodehl J. (1994). Nifedipine aggravates cyclosporine A-induced gingival hyperplasia. *Pediatric Nephrology*.

[16] Santoso C. M., Ketti F., Bramantoro T., Zsuga J., Nagy A. (2021). Association between oral hygiene and metabolic syndrome: a systematic review and meta-analysis. *Journal of Clinical Medicine*.

[17] Soskolne W. A., Klinger A. (2001). The relationship between periodontal diseases and diabetes: an overview. *Annals of Periodontology*.

[18] Pirih F. Q., Monajemzadeh S., Singh N. (2021). Association between metabolic syndrome and periodontitis: the role of lipids, inflammatory cytokines, altered host response, and the microbiome. *Periodontology 2000*.

[19] Albright J. W., Woo P. H., Ji S., Sun B., Lang K., Albright J. F. (2013). Synergism between obesity and poor oral health associated with diabetes in an elderly human population. *The Southeast Asian Journal of Tropical Medicine and Public Health*.

[20] Yang L., Zhang L., Hu J., Wang W., Liu X. (2021). Promote anti-inflammatory and angiogenesis using a hyaluronic acid-based hydrogel with miRNA-laden nanoparticles for chronic diabetic wound treatment. *International Journal of Biological Macromolecules*.

[21] el-Aassar M. R., el-Beheri N. G., Agwa M. M. (2021). Antibiotic-free combinational hyaluronic acid blend nanofibers for wound healing enhancement. *International Journal of Biological Macromolecules*.

[22] Zamboni F., Okoroafor C., Ryan M. P. (2021). On the bacteriostatic activity of hyaluronic acid composite films. *Carbohydrate Polymers*.

[23] Shirakata Y., Imafuji T., Nakamura T. (2021). Periodontal wound healing/regeneration of two-wall intrabony defects following reconstructive surgery with cross-linked hyaluronic acid-gel with or without a collagen matrix: a preclinical study in dogs. *Quintessence International*.

[24] Mamajiwala A. S., Sethi K. S., Raut C. P., Karde P. A., Mamajiwala B. S. (2021). Clinical and radiographic evaluation of 0.8% hyaluronic acid as an adjunct to open flap debridement in the treatment of periodontal intrabony defects: randomized controlled clinical trial. *Clinical Oral Investigations*.

[25] Becker L. C., Bergfeld W. F., Belsito D. V. (2009). Final report of the safety assessment of hyaluronic acid, potassium hyaluronate, and sodium hyaluronate. *International Journal of Toxicology*.

[26] Pilloni A., Zeza B., Kuis D. (2021). Treatment of residual periodontal pockets using a hyaluronic acid-based gel: a 12 month multicenter randomized triple-blinded clinical trial. *Antibiotics*.

[27] FDA (2020). *Nutrition C for FS and A. Cosmetic ingredients*.

[28] Butera A., Gallo S., Maiorani C. (2021). Management of gingival bleeding in periodontal patients with domiciliary use of toothpastes containing hyaluronic acid, lactoferrin, or paraprobiotics: a randomized controlled clinical trial. *Applied Sciences*.

[29] Chin Y. T., Tu H. P., Lin C. Y. (2021). Antioxidants protect against gingival overgrowth induced by cyclosporine A. *Journal of Periodontal Research*.

[30] Rudrapal M., Hussain N., Egbuna C. (2021). Diabetes mellitus and it management with plant-based therapy. *Dietary Phytochemicals*.

[31] Pal M., Saokar A., Gopalkrishna P., Rajeshwari H. R., Kumar S. (2020). Diode laser-assisted management of intraoral soft tissue overgrowth: a case series. *General Dentistry*.

